# Inhibition of Pneumolysin Cytotoxicity by Hydrolysable Tannins

**DOI:** 10.3390/antibiotics9120930

**Published:** 2020-12-21

**Authors:** Santeri Maatsola, Sami Kurkinen, Marica T. Engström, Thomas K. M. Nyholm, Olli Pentikäinen, Juha-Pekka Salminen, Sauli Haataja

**Affiliations:** 1 Institute of Biomedicine, Research Center for Cancer, Infections and Immunity, University of Turku, 20014 Turku, Finland; sjjmaa@utu.fi; 2 Institute of Biomedicine, Integrative Physiology and Pharmacology, University of Turku, 20014 Turku, Finland; sami.t.kurkinen@utu.fi (S.K.); olli.pentikainen@utu.fi (O.P.); 3 Natural Chemistry Research Group, Department of Chemistry, University of Turku, 20500 Turku, Finland; mtengs@utu.fi (M.T.E.); j-p.salminen@utu.fi (J.-P.S.); 4 Institute of Biomedicine, Bioanalytical Laboratory, University of Turku, 20014 Turku, Finland; 5 Biochemistry Faculty of Science and Engineering, Abo Akademi University, 20500 Turku, Finland; Thomas.Nyholm@abo.fi

**Keywords:** *Streptococcus pneumoniae*, pneumolysin, tannins, pentagalloylglucose, gemin A

## Abstract

*Streptococcus pneumoniae* causes invasive infections such as otitis media, pneumonia and meningitis. It produces the pneumolysin (Ply) toxin, which forms a pore onto the host cell membrane and has multiple functions in the pathogenesis of *S. pneumoniae*. The Ply C-terminal domain 4 mediates binding to membrane cholesterol and induces the formation of pores composed of up to 40 Ply monomers. Ply has a key role in the establishment of nasal colonization, pneumococcal transmission from host to host and pathogenicity. Altogether, 27 hydrolysable tannins were tested for Ply inhibition in a hemolysis assay and a tannin-protein precipitation assay. Pentagalloylglucose (PGG) and gemin A showed nanomolar inhibitory activity. Ply oligomerization on the erythrocyte surface was inhibited with PGG. PGG also inhibited Ply cytotoxicity to A549 human lung epithelial cells. Molecular modelling of Ply interaction with PGG suggests that it binds to the pocket formed by domains 2, 3 and 4. In this study, we reveal the structural features of hydrolysable tannins that are required for interaction with Ply. Monomeric hydrolysable tannins containing three to four flexible galloyl groups have the highest inhibitory power to Ply cytotoxicity and are followed by oligomers. Of the oligomers, macrocyclic and C-glycosidic structures were weaker in their inhibition than the glucopyranose-based oligomers. Accordingly, PGG-type monomers and oligomers might have therapeutic value in the targeting of *S. pneumoniae* infections.

## 1. Introduction

*Streptococcus pneumoniae* is a major cause of human pneumonia, otitis media and meningitis. It is estimated that it causes over one million deaths globally every year [1]. Due to the emergence of antibiotic resistance and rapid increase in non-vaccine serotypes, the global burden of *S. pneumoniae* infections remains a serious problem [2,3]. This calls for the development of alternative non-bacteriosidic compounds, which would prevent infections. Compounds that target bacterial in vivo virulence factors essential for survival in the host, such as pneumolysin (Ply), are candidates that could be used in conjunction with other antibiotics or alternative antimicrobials. Tannin, tellimagrandin II, has been found to enable the effective dose of current antibiotics to be reduced in the treatment of MRSA strains of *Staphylococcus aureus* [4]. It serves as an example of the potential use of tannins as alternatives to antibiotics or to be used in conjunction with antibiotics against multi-resistant bacteria.

Several virulence factors important in adhesion, colonization and pneumonia have been studied in mouse models. Pneumococcal surface protein C (PspC), also designated as choline binding protein A (CbpA,) and pneumococcal surface protein A (PspA) were shown, in a mouse model, to be required for nasopharyngeal colonization and pneumonia [5]. Ply was also required for full *S. pneumoniae* virulence in a pneumonia model. Mutants of Ply have been studied in a mouse intraperitoneal challenge model [6,7]. Interestingly, Ply point mutations abolishing its cytotoxicity and complement activating properties were still virulent, whereas a Ply deletion mutant was significantly attenuated. It suggests that Ply has other functions that are also required in pneumococcal virulence. The expression levels of Ply can vary between strains of different serotypes [8]. However, this does not correlate to pneumococcal virulence, which is more dependent on serotype and genetic background of the pneumococcal strains. The virulent, bioluminescent, serotype two pneumococcal strain D39Xen7 has been used for in vivo bioluminescent imaging (BLI) in a mouse model [9]. BLI was used to follow the replication of *S. pneumoniae cbpA*, *pspA*, *Ply*, pyruvate oxidase (*spxB*), autolysin (*lytA*) and neuraminidase A (*nanA*) mutants in mice using intranasal, intratracheal and intravenous challenges. This study showed the important role of Ply in *S. pneumoniae* growth in lung, blood and brain. In addition, the production of hydrogen peroxide, the other pneumococcal toxin synthesized by SpxB, was shown to contribute to nasopharyngeal colonization and bacterial growth in lung. *CbpA* was shown to be required for nasopharyngeal colonization and for invasion of pneumococci into the brain. Numerous studies have shown that *S. pneumoniae* has multiple virulence factors which contribute to its adhesion to host nasopharyngeal mucosal surfaces, its escape from immune defense mechanisms and its invasion into host tissues, as reviewed by Weiser et al. [10].

Pneumolysin is a 53-kDa protein of *S. pneumoniae* belonging to the family of cholesterol-dependent cytolysins (CDC) [11]. Upon interaction with eukaryote membrane cholesterol, pneumolysin is oligomerized, and it creates a pore onto the membrane and kills the target cell. It is composed of four distinct domains. Domains 1, 2, and 3 are required for oligomerization and domain 4 is responsible for host cell recognition and cholesterol binding [12]. Unlike the other members of the CDC toxin family, Ply has no signal peptide for secretion and is released from the bacterial cytoplasm by autolysis and allolysis during the natural competence for DNA transformation [13]. Ply has also been shown to be secreted by the accessory SecY2A2 Sec system and is found in the active form attached to the pneumococcal cell wall [14,15]. Ply is a highly multifunctional toxin, which, in addition to its hemolytic and cytolytic activity, can cause pyroptosis and apoptosis, regulate complement activity, induce a number of signaling effects and also play a role in *S. pneumoniae* transmission from host to host [16,17,18]. Since Ply contributes to *S. pneumoniae* survival throughout its presence in the host (i.e., colonization, infection and transmission), it is an important target for the prevention of pneumococcal infections.

Tannins can be classified into condensed tannins (syn. proanthocyanidins), hydrolysable tannins and phlorotannins as reviewed by Quideau et al. [19]. They are highly versatile plant polyphenols, important to host defense mechanisms against pathogens and as scavengers of UV-B light and highly reactive oxidative radicals [19]. Functionally, tannins can react with proteins by binding with them as multidentate ligands, ultimately leading to the precipitation of tannin–protein complexes, thereby reducing the nutritional value of plants to pathogens. Phenolic rings can interact with proteins by forming hydrophobic interactions by π-stacking with aromatic side-chains of proteins and via van der Waals interactions. Phenolic hydroxy groups can interact by forming hydrogen bonds and dipole–dipole interactions.

Recently, epigallocatechin gallate (EGCG) was shown to inhibit pneumolysin hemolysis and cytotoxicity [20]. This compound is a gallic acid ester of a flavan-3-ol monomer. Interestingly, all hydrolysable tannins consist of this same gallic acid moiety esterified typically to a sugar-type polyol and are modified further by, e.g., oxidative reactions to yield almost 1000 monomeric and oligomeric structures. All these can be purified and studied as individual compounds, unlike proanthocyanidins and phlorotannins that are typically complex mixtures of their polymeric forms [21,22,23]. Here, we purified 27 hydrolysable tannins and studied how they could inhibit Ply cytotoxicity. We show that the hemolysis, Ply oligomerization and cytotoxicity of Ply is inhibited by hydrolysable tannins. The structure–activity relationship suggests that the polyvalency and flexibility of the galloyl groups and the formation of flexible oligomers increase the inhibitory power of these tannins.

## 2. Results

### 2.1. Inhibition of Hemolysis by Hydrolysable Tannins

**Figure 1:**
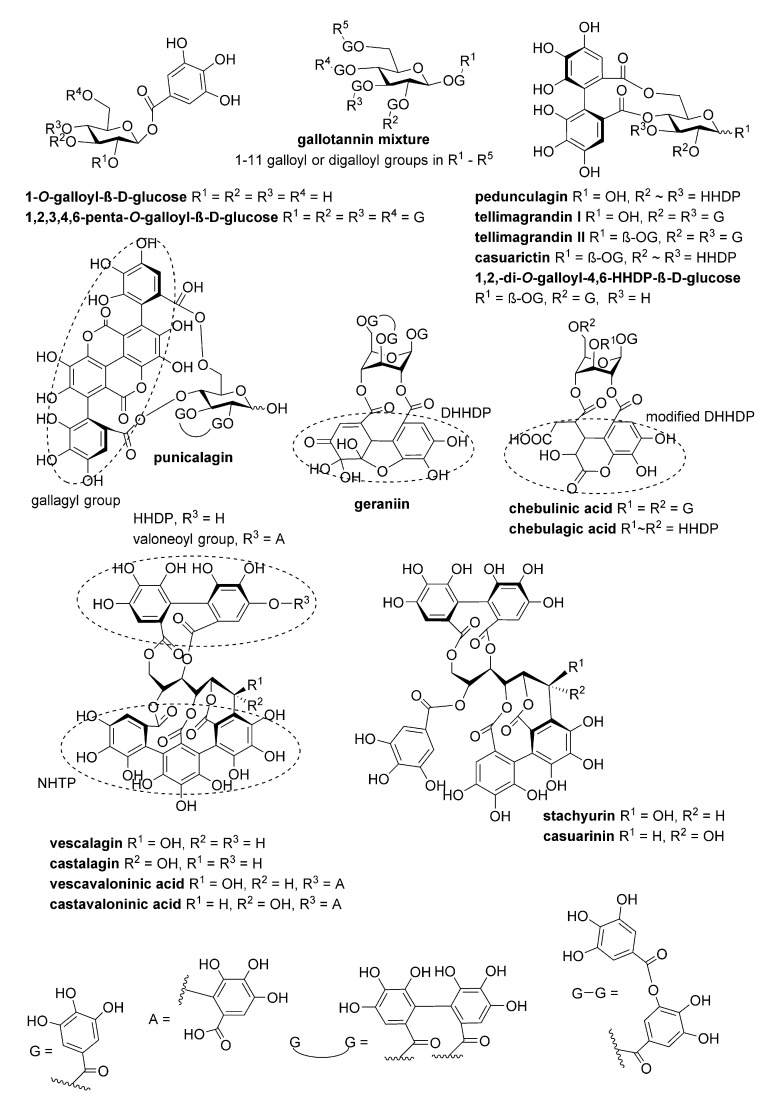
The structures of the monomeric tannins used in this study.

**Figure 2:**
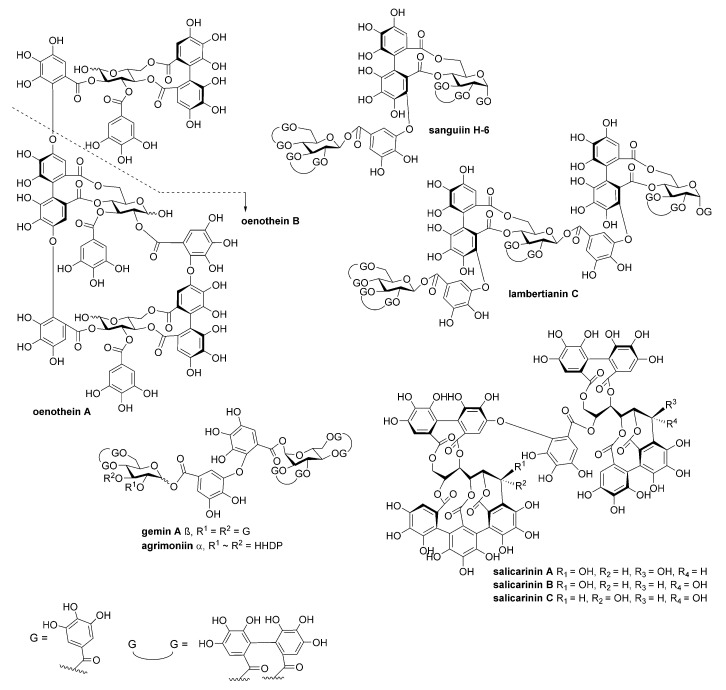
The structures of the oligomeric tannins used in this study.

Twenty-seven hydrolysable tannins (Figure 1 and Figure 2, Appendix A Table A1) were tested for their inhibitory activity against pneumolysin hemolytic activity. An inhibition assay performed with two-fold dilutions of tannins enabled us to classify the compounds into three groups: negative or weak inhibitors (IC_50_ ≥ 100 nM), moderate inhibitors (IC_50_ 40–90 nM) and potent inhibitors (IC_50_ 10–30 nM). For the monomers, at least four flexible galloyl groups or three galloyl groups and one hexahydroxydiphenoyl (HHDP) group were critical to the activity, while for oligomers, both flexibility and the presence of a glucopyranose-based core instead of an acyclic one were important. The non-flexible macrocyclic oenothein B and the C-glycosidic oligomers salicarinins A, B and C were the weakest of the oligomers. PGG (1,2,3,4,6-penta-*O*-galloyl-β-D-glucose), the most active monomer, and gemin A, the most active oligomer, were chosen for more extensive characterization of the inhibitory properties of these compounds. The moderate and weak inhibitors oenothein B (oligomer) and vescalagin (monomer) were chosen as control compounds.

### 2.2. Inhibitory Power of PGG and Gemin A

**Figure 3:**
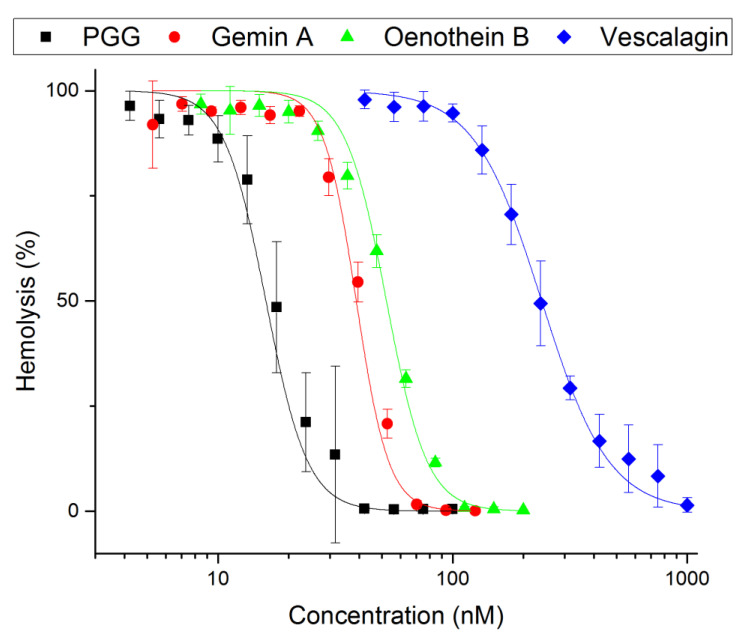
The inhibition of pneumolysin activity by tannins. The tannins used were the monomeric tannins 1,2,3,4,6-penta-*O*-galloyl-β-D-glucose (PGG) and vescalagin; and the oligomeric tannins gemin A and oenothein B. The hemolysis caused by pneumolysin (Ply) was analyzed based on the release of hemoglobin from the lysed erythrocytes. The hemoglobin was quantitated based on its absorbance at 570 nm (triplicate measurements). The % hemolysis values are shown in the *y*-axis as a function of inhibitor concentration (*x*-axis). Error bars represent standard deviation of triplicate measurements. IC_50_ values for representative polyphenols were calculated with logistic fit using Origin.

**Figure 4:**
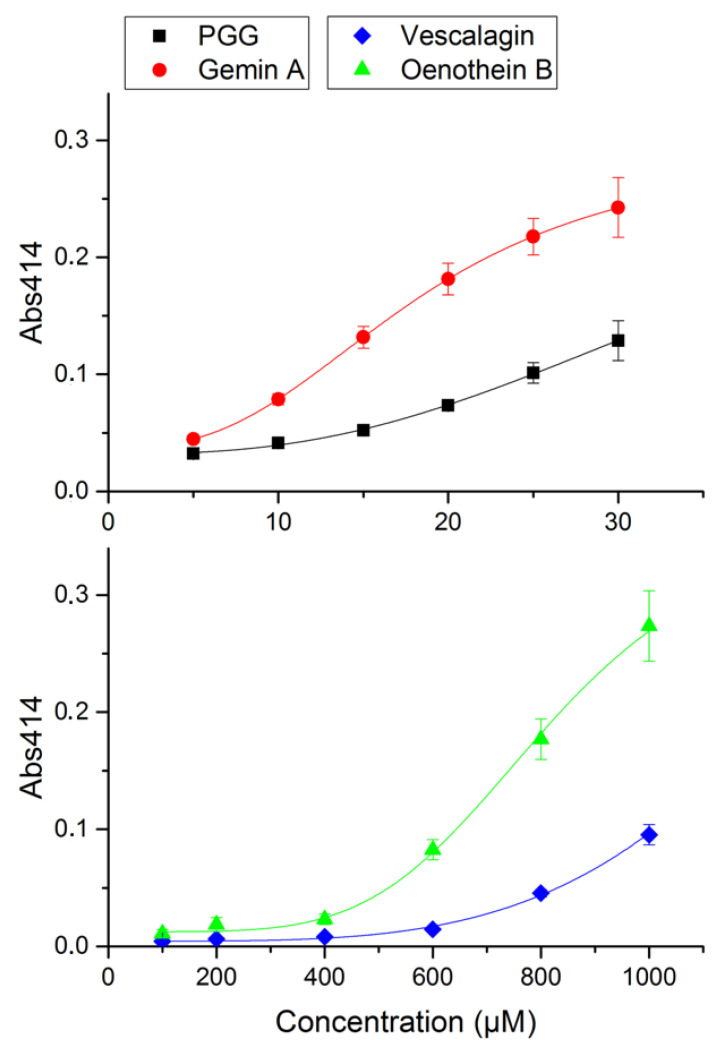
The precipitation assay of pneumolysin–tannin interaction. The tannins used were the monomeric tannins PGG and vescalagin; and the oligomeric tannins gemin A and oenothein B. The precipitation caused by tannins was analyzed by measurement of the absorbance at 414 nm. The minimum concentration still precipitating Ply was calculated. Error bars represent standard deviation of six replicate measurements. The data were analyzed with logistic fit using Origin.

The tannin concentrations giving 50% inhibition in the hemolysis assay (IC_50_) were determined (Figure 3). PGG had an IC_50_ of 18 ± 0.7 nM and an inhibitory power of 13 compared to vescalagin (IC_50_ of 240 ± 5.3 nM and an inhibitory power of 1). The inhibitory power of gemin A was 5.9, and that of oenothein B was 4.4 (IC_50_ 41 ± 1 nM and 55 ± 2.1 nM, respectively). The most potent compound causing precipitation was gemin A. Its potency to cause precipitation was 130-fold higher compared to vescalagin. In contrast, PGG was two-fold weaker compared to gemin A but was still 58-fold stronger compared to vescalagin (Figure 4).

The hemolysis inhibition and precipitation assay results were compared (Table 1). The results show that the precipitation required a 770-fold higher concentration of PGG and a 150-fold higher concentration of gemin A compared to the hemolysis IC_50_ concentrations. The moderate inhibitor oenothein B and weak inhibitor vescalagin required 9300-fold and 3400-fold higher precipitating concentrations, respectively, compared to their IC_50_ values.

### 2.3. PGG Abolishes Oligomerization of Pneumolysin

**Figure 5:**
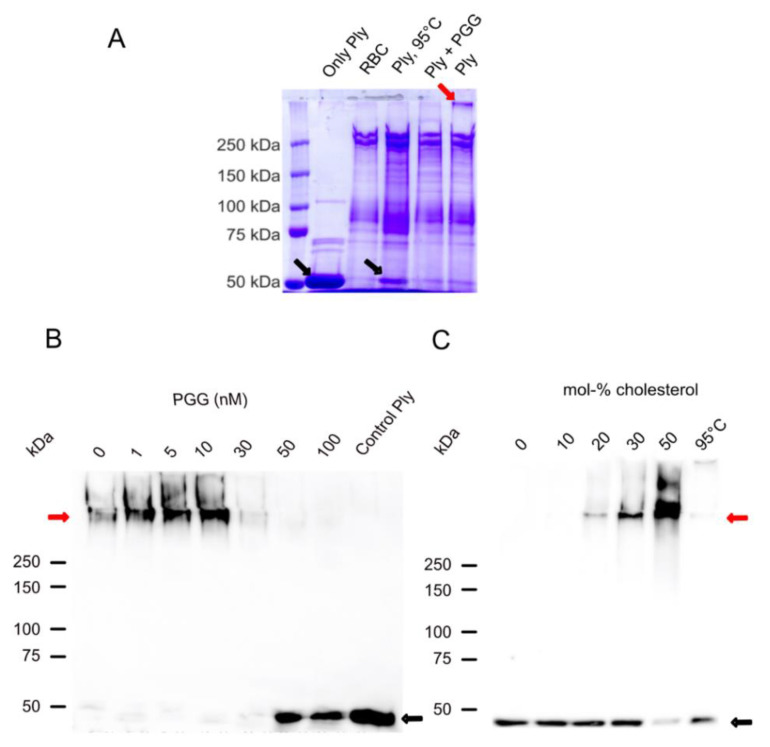
**Pneumolysin oligomerization assay using erythrocytes.** (**A**) Coomassie blue-stained SDS-PAGE gel of erythrocyte membrane proteins and oligomerized Ply. Samples: Ply without incubation with erythrocytes, monomeric Ply; RBC, a control sample of erythrocytes incubated without Ply; Ply 95 °C, erythrocytes incubated with Ply and the sample was heated at 95 °C to depolymerize Ply oligomers; Ply + PGG, erythrocytes were mixed with Ply preincubated with PGG; Ply, erythrocytes incubated with Ply only. (**B**) Inhibition of Ply oligomerization by dilution series of PGG. Pneumolysin oligomers were detected with Western blotting using an anti-His antibody. Samples: MonoPly, a control of Ply without incubation with erythrocytes; Ply oligomers in the presence of PGG inhibitor concentrations (0–100 µM). (**C**) Control experiment showing cholesterol dependence of Ply oligomerization using liposomes. Liposomes containing 0–50 mol-% of cholesterol were incubated with Ply, and the liposomes were solubilized into SDS-PAGE sample buffer, and Ply was detected with Western blotting as in (**B**). Red arrows indicate oligomerized Ply and black arrows monomeric Ply.

The proteins were separated on SDS-PAGE gel (6% gel). The monomeric Ply has an apparent MW of 55 kDa, whereas the oligomerized Ply runs as a high molecular weight complex migrating just underneath the separating gel (Figure 5A). As can be seen from the Coomassie-stained gel, the Ply complex was abolished with PGG and boiling at 95 °C. A sample containing only Ply contained the 55-kDa monomeric form. To reveal the minimum amount of PGG that can inhibit Ply oligomerization, dilution series of PGG were incubated with Ply, and the oligomerization was detected with SDS-PAGE along with detection of Ply with Western blotting using the anti-His antibody (Figure 5B). The concentration that still inhibited Ply oligomerization was 30 µM. As a control for the dependence on the membrane cholesterol for Ply oligomerization, liposomes with varying mol-% (0–50) of cholesterol were incubated with Ply (Figure 5C). The results show that increasing concentrations of cholesterol above 20 mol-% induced Ply oligomerization.

### 2.4. Inhibition of Ply Cytotoxicity by PGG

**Figure 6:**
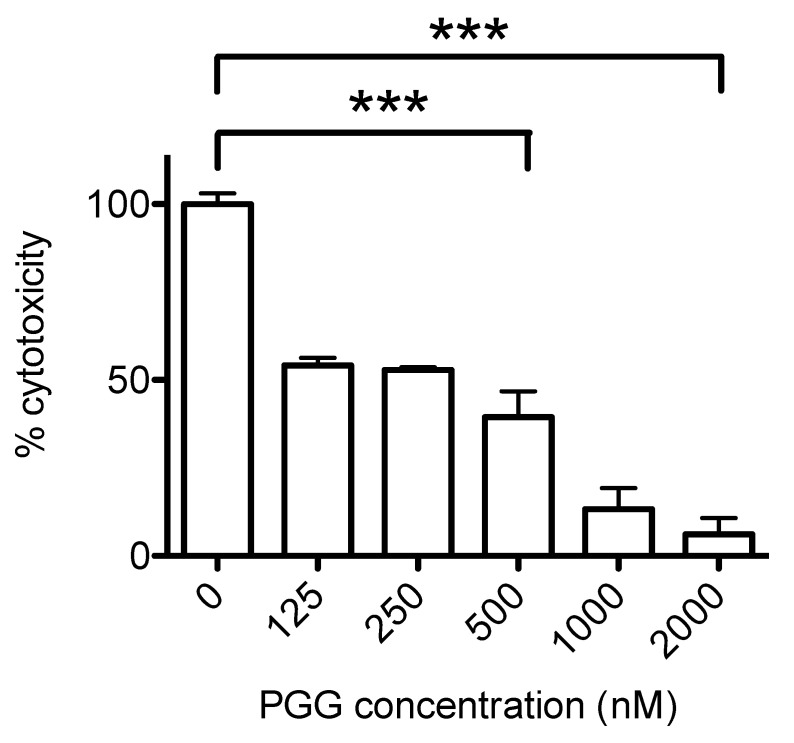
The inhibition of pneumolysin cytotoxicity with PGG. The cytotoxicity of Ply in the presence of PGG was quantitated with LDH cytotoxicity assay. The normalized LDH activities are shown as % cytotoxicity compared to Ply without inhibitor. The error bars represent standard deviation of triplicate determinations. Statistical significance, shown for PGG inhibitor concentrations of 500 and 2000 nM, was analyzed with a one-way ANOVA with Tukey’s multiple comparison test (three asterisks *p* ≤ 0.001). The statistics were done with Prism 4 (GraphPad).

The Ply cytotoxicity to lung pneumocyte A549 cells was analyzed by incubation of 2 nM of Ply with the cells grown in plastic wells (Figure 6). The cells remained bound to the wells during Ply treatment. The inhibition of PGG to pneumolysin cytotoxicity measured by lactate dehydrogenase (LDH) release by dead cells was measured as described in the methods. Lower concentrations of PGG (≤250 nM) caused a moderate inhibition, whereas 500, 1000 and 2000 nM of PGG inhibited LDH release by 60%, 87% and 90%, respectively, compared to the cells incubated with pneumolysin alone.

### 2.5. In Silico Studies of PGG Interaction with Ply

**Figure 7:**
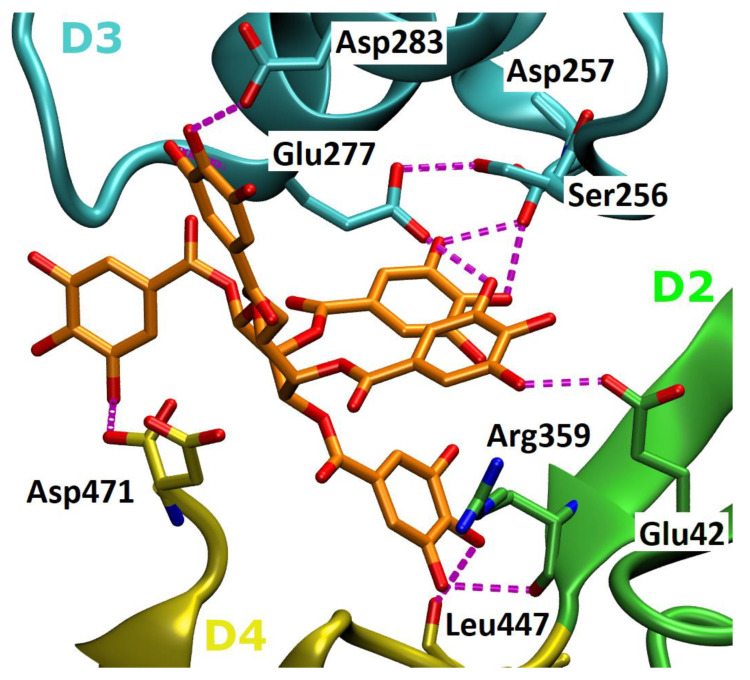
**The binding mode of Ply-PGG.** PGG (orange) fits in the cleft between domains 2 (D2, green), 3 (D3, cyan) and 4 (D4, yellow) of Ply. Possible hydrogen bond interactions are shown as dashed, purple lines. The binding orientation represents the docking conformation after energy minimization steps. The figure was prepared with Visual Molecular Dynamics (VMD) 1.9.2 [25].

As suggested in previous studies with epigallocatechin gallate (EGCG) and verbascoside, the cleft between domains 2, 3 and 4 is considered an important target to block Ply oligomerization [20,24]. Molecular modeling suggests that PGG also binds to this cleft (Figure 7). After energy minimization steps, some key amino acids in interactions are Glu42, Ser256, Asp257, Glu277 and Arg359 that hydrogen-bond to PGG. Similar interactions are also reported for EGCG and verbascoside, which were suggested to interact, e.g., with Ser256, Glu277, Tyr358 and Arg359 in a former study [20,24]. After the simulation of 50 ns, PGG moved further from Asp257 and Ser256 but was still interacting with Glu42, Glu277 and Arg359.

## 3. Discussion

In the present study, we have evaluated the structure–function relationship of hydrolysable tannins for their inhibitory activity towards pneumolysin, the virulence factor of *Streptococcus pneumoniae*. These results suggest that the molecular mechanisms of tannin interaction with Ply and inhibition of the oligomerization quite closely followed the protein precipitation capacity (PPC) patterns earlier detected for these tannins using bovine serum albumin (BSA) as a model protein [22]. The molecular weight, number of galloyl groups, degree of oxidative coupling between the galloyls, positional isomerism and cyclic vs. acyclic glucose core were the major structural features affecting the PPC of the monomeric hydrolysable tannins with BSA. Regarding the PPC, oligomers were superior to monomers and the PPC of the oligomers was less affected by the functional groups and more so by their size and overall flexibility. The PPC of tannins with Ply observed in this study is reminiscent of the precipitation results obtained with BSA protein [22], suggesting that the structure–activity patterns found in that study for 32 purified hydrolysable tannins could be valid against Ply as well as BSA.

The Ply oligomerization assay was developed using erythrocytes as target cells. The use of erythrocytes was chosen since recent findings show that cell surface carbohydrates are also required for Ply oligomerization and pore formation [26,27]. The formation of a large 2500-kDa Ply complex was detected in Western blotting. The separation of very high-molecular-weight Ply oligomers is at the limit of the resolution capacity of polyacrylamide gel electrophoresis. However, by using the ability of oligomerized Ply to resist mild denaturing conditions and its depolymerization into the monomer by stronger denaturation conditions (incubation at 95 °C), we were able to confidently detect the high-molecular-weight Ply oligomer using Western blotting. Incubation of Ply with PGG abolished oligomerization, as shown in Figure 5A. The reduced signal of Ply at the concentration of 30 nM of PGG (Figure 5B) could be due to the formation of larger soluble complexes that are out of the separation range using polyacrylamide gel electrophoresis. Similarly, the formation of larger soluble complexes of Ply with cholesterol-containing liposomes could explain the second high molecular weight band in the control experiment with Ply and liposomes containing 50 mol-% cholesterol (Figure 5C). Higher concentrations of PGG abolish oligomerization and the monomeric Ply can be detected as a 55-kDa band in the Western blot.

According to computational studies, the increased inhibitory power of PGG is based on pentameric rotatable galloyl groups (Figure 7). Previously, epigallocatechin gallate (EGCG) was shown to inhibit Ply hemolytic activity; however, micromolar concentrations were required for inhibition [13], which could be due to the lower number of free galloyl groups and, thus, the weaker tannin nature of EGCG. This could also explain why vescalagin has a much higher IC_50_ concentration for hemolysis inhibition than PGG, although they have the same number of aromatic rings. In PGG, all galloyls are freely rotating and flexible, while in vescalagin, two of the galloyls are C-C linked to form a rigid HHDP group and the other three galloyls are linked to form a rigid nonahydroxytriphenoyl NHTP group (Table 1, Figure 1). In contrast, gemin A and oenothein B have two galloyl groups not bonded together, enabling a better binding affinity than what is found in vescalagin (Figure 2). Oligomers, in general, are known to bind proteins more efficiently than monomers [22]. The macrocyclic dimer oenothein B loses part of its potency since the monomers are linked by two bonds, thus losing the flexibility between the monomers. It is possible that the flexible dimer gemin A could be able to bind to two Ply molecules, which would explain its better potency in causing precipitation in comparison to PGG and oenothein B.

Pneumolysin can induce cell death by three different pathways, e.g., apoptosis, programmed necroptosis, pyroptosis and direct cytotoxicity, in a cell-type-specific manner [17,28]. Direct cytotoxicity of Ply released from *S. pneumoniae* has been shown to cause cell death and LDH release of A549 cells [29]. The cells targeted by Ply have been shown to try to repair their cell membrane by the formation of Ply containing nanotubes, which subsequently form microvesicles that are shed by the membrane. [30]. Cell death is eventually caused by the leakage of calcium into the cell cytoplasm. Further studies will be required to clarify the mechanisms of PGG in inhibiting the cellular death mechanisms by Ply.

Tannins are secondary metabolites of plants which have been used as beneficial nutraceuticals [31]. Accordingly, purified tannins have been studied for their health-promoting properties and as candidates for development of therapeutic compounds, as reviewed by Quideau et al. [19]. PGG has been found to inhibit growth of *Acinetobacter baumannii* and the cytotoxic concentrations tested with keratinocyte cells suggest that the effective tissue concentrations that could inhibit Ply would be tolerated and not be harmful to humans [32]. The potent inhibitors PGG, gemin A and tellimagrandin II (Table A1) could be used in conjunction with other antimicrobials in preventing pneumococcal infections in the upper respiratory tract. Therefore, tannins are promising compounds that could be used together with other compounds that inhibit pneumolysin activities.

## 4. Materials and Methods

### 4.1. Tannins

The structures of the polyphenols studied are presented in Figure 1 and Figure 2. They were purified and characterized as described in Engström et al. (2019). The compounds were dissolved to 10% (*v*/*v*) in an ethanol–water solution.

### 4.2. Cloning and Expression of Recombinant Pneumolysin

Pneumolysin gene was cloned with primers gacgacgacaagatggcaaataaagcagtaaatgac and gaggagaagcccggttta ctagtcattttctaccttatc. The DNA fragment was amplified with Phusion HotStart II DNA polymerase and was cloned into ligation independent cloning (LIC) vector pET46EkLIC (Novagen), and the cloned insert was verified with sequencing. The expression plasmid was transformed into *E. coli* BL21(DE3) for protein expression. The His-tagged recombinant protein was purified using Ni-NTA affinity chromatography and gel filtration with a HiLoad 16/60 Superdex column as described before [33]. Briefly, the expression of His-tagged Ply was induced with 1 mM isopropylthiogalactoside in Luria-Bertani broth (100 μg/mL ampicillin). Bacteria were lysed with lysozyme in the presence of a protein inhibitor cocktail. The lysate was sonicated, cleared with centrifugation and filtered. The recombinant Ply was affinity purified and the final purification was done with gel filtration using Tris-Cl, pH 7.5, 0.15 M NaCl running buffer. The purity of the recombinant protein was analyzed with SDS-PAGE chromatography.

### 4.3. Hemolysis Inhibition Assays with Tannins

Native human erythrocytes were taken into EDTA tubes. The erythrocytes were washed three times with PBS (0.15 M NaCl, 2.7 mM KCl, 8.1 mM Na_2_HPO_4_ and 1.5 mM KH_2_PO_4_) by centrifugation at 700× *g* for 10 min. The purified erythrocytes were stored at +4 °C. Erythrocytes (1% *v*/*v*) in PBS were mixed with 1 nM Ply and dilution series of polyphenol inhibitors in 96-well microtiter plates. The reaction was incubated for 30 min at 37 °C. The plates were centrifuged, and the supernatant was pipetted into flat-bottomed microtiter plates to measure the absorbance at 570 nm (VICTOR X4, PerkinElmer, Turku, Finland) of the released hemoglobin from lysed erythrocytes. The absorbances of triplicate measurements are presented as a function of inhibitor concentration. The IC_50_ values were calculated with Origin data analysis software (2016, OriginLab Corporation, Northampton, MA, USA).

### 4.4. Precipitation Assay

The precipitation assay with tannins was done as described previously with bovine serum albumin [22]. First, 10 μM of Ply and dilution series of polyphenols (5–1000 µM) were incubated in a Na-acetate buffer (200 mM NaCl and 50 mM CH_3_COONa; pH 5.0) at 25 °C for 30 min with shaking. The reactions were carried out in Multiskan Ascent plate reader (Labsystems, Vantaa, Finland) and the precipitation was measured at 414 nm. The minimum concentration precipitating Ply was calculated with Origin data analysis software (2016, OriginLab Corporation, Northampton, MA, USA).

### 4.5. Liposomes

Vesicles were prepared by dissolving 1 μM 1-palmitoyl-2-oleoyl-glycero-3-phosphocholine (POPC, Avanti Polar lipids) and 1–50 mol-% cholesterol into chloroform: methanol (2:1, vol/vol). The solvent was evaporated with nitrogen, and the lipid film was hydrated into 10 mM Tris-Cl and 140 mM NaCl buffer for 30 min at 60 °C to a final concentration of 1000 μM. The lipid-buffer suspension was briefly vortexed followed by the extrusion procedure according to the manufacturer’s instructions (Avanti mini extruder using 0.1 μm polycarbonate membranes filter (Avanti Polar Lipids, Alabaster, AL, USA)) to form large unilamellar vesicles [34].

### 4.6. Oligomerization Assay

For the oligomerization assay, 100 nM Ply (3 µg), 1% (*v*/*v*) erythrocytes and 100 µM PGG in PBS were incubated for 2 min at 25 °C and were centrifuged at 16,000× *g* for 30 min at +7 °C. The pellet containing intact erythrocytes and the membranes from lysed cells were suspended into the water to complete the lysis and recentrifuged at 16,000× *g* for 30 min. The membranes were suspended into an SDS-PAGE sample buffer (60 mM Tris-HCl, pH 6.8; 2% SDS; 0.001% bromophenol blue; 0.5% β-mercaptoethanol) without incubation at 95 °C and the proteins were separated in 6% SDS-PAGE gel. The gel was stained with Coomassie blue (40% methanol; 10% acetic acid; 0.25% Brilliant blue R-250) and destained in 20% methanol and 5% acetic acid overnight. The proteins were transferred to polyvinyldifluoride (PVDF) membrane for 30 min, 25 V in SDS-PAGE running buffer (25 mM Tris, 190 mM glycine, 0.1% SDS) using the Trans-Blot Turbo Transfer System (Bio-Rad Laboratories Inc., Hercules, CA, USA). The membrane was blocked with 5% (*w*/*v*) defatted milk powder in TBS-Tween (10 mM Tris pH 7.5; 150 mM NaCl; 0.05% Tween 20) for 30 min at 25 °C. The His-tagged Ply was detected with anti-His antibody (1:5000 dilution, Sigma-Aldrich, St. Louis, MO, USA) and horseradish peroxide-labelled anti-mouse antibody (1:10,000 dilution, DakoCytomation, Glostrup, Denmark). After washing, the membrane was incubated with ECL detection substrate (WesternBright Quantum, Labtech, Heathfield, UK) and was imaged using ImageQuant LAS 4000 (GE Healthcare Bio-Sciences AB, Uppsala, Sweden).

### 4.7. A549 Cell Cytotoxicity Assay

For the A549 cell cytotoxicity assay, 5000 cells/well of A549 cells were grown in Dulbecco’s modified Eagle Medium, DMEM (Thermo Fischer Scientific Inc., Waltham, MA, USA), 2 mM glutamine and 10% fetal calf serum (FCS) for 48 h in 96-well microtiter plates. Cells were washed with PBS and dilutions of PGG (0, 125, 250, 500, 1000 and 2000 nM) and 2 nM Ply in PBS were added to the wells and they were incubated for 2 h at 37 °C in 5% CO_2_ atmosphere. Ply cytotoxicity was determined with the Cytotoxicity Detection Kit^PLUS^ (Roche Diagnostics GmbH, Mannheim, Germany) according to the manufacturer’s instructions. The released LDH was measured at A_490_. Complete lysis control was measured in the presence of 0.02% Triton X-100, and for background measurements, cells were incubated without Ply. The values were normalized by calculating using the equation

(A_490 Ply+inhibitor_ − A_490 Background_)/(A_490 Triton X-100_ − A_490 Background_) (1)

### 4.8. Molecular Modelling

PGG was converted to 3D SYBYL MOL2 format containing OPLS3 partial charges with LIGPREP in MAESTRO 2018-1 (Schrödinger, LLC, New York, NY, USA, 2018) and considering the protonation at pH 7.4. Target structure preparation of the Ply X-ray structure (PDB code 5CR6 [12]) was performed using Protein Preparation Wizard in MAESTRO at pH 7.4 using default settings. Extra Precision (XP) mode of GLIDE [35,36] was used to dock PGG between Ply domains 2, 3 and 4 (centroid coordinates 4.337, 21.215, 220.177).

For molecular dynamic simulations, PGG was parametrized with ANTECHAMBER 17 [37] using the AM1-bcc charge method. Tleap in the AMBER 18 package (University of California, San Francisco, Case et al., 2018) was used to parametrize protein with ff14SB force field [38], combine protein–ligand complex, add hydrogen atoms and solvate the system with a rectangular box of transferable intermolecular potential three-point (TIP3P) water molecules [39] extending 13 Å in every direction around the solute. The system was neutralized by adding Na+ counter ions. A 50-ns simulation was run with NAMD2.13 [25,40] in four steps: (1) 15,000 steps of energy minimization with restrained Ca atoms (5 kcal/mol); (2) 15,000 steps of energy minimization without restraints; (3) 180,000 steps of molecular dynamics simulation with restrained Ca atoms (5 kcal/mol); (4) 25,000,000 (50 ns) steps of simulation without restraints.

## 5. Conclusions

In summary, we have shown the specific inhibition of Ply by hydrolysable tannins containing galloyl groups using cellular models, i.e., inhibition of Ply-induced hemolysis and cytotoxicity to A549 cells. The structure–function relationship of Ply–tannin interaction was determined by hemolysis inhibition and by the direct precipitation of Ply by tannins. The interaction of Ply with PGG was modelled with docking experiments. The efficient and specific inhibition by PGG is suggested to rely on the interaction between the phenolic hydroxy groups of PGG and Ply polar amino acids situated in the pocket formed by domains 2, 3 and 4. PGG abolished the Ply complex formation on the surface of erythrocytes, as shown by Western blotting of the solubilized erythrocyte membranes treated with recombinant Ply. The structural features reveal that (1) hydrolysable tannin monomers such as PGG containing polyvalent and flexible galloyl groups as well as (2) galloyl-containing oligomers consisting of glucopyranose-based monomers with non-macrocyclic linkage have the strongest inhibitory powers and are, thus, potential therapeutic compounds for inhibiting Ply cytotoxicity.

## Figures and Tables

**Table 1:** Comparison of the hemolysis inhibition and tannin precipitation powers of selected hydrolysable tannins.

Compound	Hemolysis Inhibition		Minimum Precipitating Concentration	
	IC_50_ [nM]	Inhibitory Power ^a^	(µM)	Relative Power
PGG	18 ± 0.7	13	14	58
Gemin A	41 ± 1.0	5.9	6.2	130
Oenothein B	55 ± 2.1	4.4	510	1.6
Vescalagin	240 ± 5.3	1	820	1

^a^ Inhibitory powers were obtained by dividing the IC_50_ of the compound with IC_50_ of vescalagin.

## Appendix A

**Table A1:** Screening of tannin compounds with hemolysis assays. IC_50_ values were estimated from the inhibition curves. Compounds taken for more detailed analysis are highlighted with yellow.

Compound	MW (Da)	IC_50_ (nM)
1,2,3,4,6-penta-*O*-galloyl-β-D-glucose	940.67	10
Tellimagrandin II	938.66	10
Gemin A	1873.28	20
Lambertianin C	2805.9	20
Sanguiin H-6	1871.27	20
Agrimoniin	1871.27	30
Gallotannin mixture	1396.99 *	40
Casuarictin	936.64	40
Oenothein A	2353.62	40
Salicarinin B	1869.25	50
Oenothein B	1569.08	60
Tellimagrandin I	786.55	60
Punicalagin	1084.71	60
1,2,-di-*O*-galloyl-4,6-HHDP-β-D-glucose	786.55	70
Salicarinin A	1869.25	80
Castalagin	934.63	80
Casuarinin	936.64	90
Geraniin	952.64	100
Salicarinin C	1869.25	100
Stachyurin	936.64	>100
Pedunculagin	784.54	>100
Vescalagin	934.63	>100
Castavaloninic acid	1102.73	>100
Chebulagic acid	954.66	>100
Vescavaloninic acid	1102.73	>100
Chebulinic acid	956.67	>100
1-*O*-galloyl-β-D-glucose	332.26	>100

* Molecular weight of the gallotannin mixture presented based on octagalloylglucose.
